# Challenges, Recommendations, and Epidemiology of Pulmonary Embolism in India: A Narrative Review

**DOI:** 10.7759/cureus.64195

**Published:** 2024-07-09

**Authors:** Sadanand M Shetty, Agam Vora, Robbie George, Vidita M

**Affiliations:** 1 Cardiology, Karamshibhai Jethabhai Somaiya Super Specialty Institute, Mumbai, IND; 2 Pulmonology, Vora Clinic, Mumbai, IND; 3 Department of Vascular and Endovascular Surgery, Narayana Institute of Vascular Sciences, Bangalore, IND; 4 Internal Medicine, Pfizer Ltd, Mumbai, IND

**Keywords:** pulmonary artery, penumbra aspiration system, non-vitamin k antagonist oral anticoagulants, deep vein thrombosis, computed tomography pulmonary angiogram, chronic obstructive pulmonary disease, cardiopulmonary disorders, anticoagulation

## Abstract

An embolized clot that travels to the lungs from the legs or, less commonly, other parts of the body (known as deep vein thrombosis or DVT) causes pulmonary embolism (PE), which is characterized by obstruction of blood flow to the pulmonary artery. As PE has the propensity to masquerade as various illnesses affecting both the cardiovascular (CV) and the respiratory system, it is crucial to identify PE at the earliest. Appropriate diagnosis of PE may lead to earlier treatment and improved patient outcomes. While pulmonary angiography remains the established gold standard for diagnosing PE, the contemporary standard of care for this condition is the computed tomography pulmonary angiogram (CTPA). Anticoagulation therapy is the fundamental strategy for managing PE, with the forefront of treatment being the use of novel and upcoming oral anticoagulants known as non-vitamin K antagonist oral anticoagulants (NOACs). The NOACs provide a practical single-drug treatment strategy, which does not hinder the patient’s lifestyle and domestic responsibilities. Although PE may be fatal, early detection may lead to effective management. Despite that, mortality and morbidity associated with PE are very high in India. The awareness among Indian healthcare professionals about PE should be improved, and unified pan-country diagnostic and management guidelines should be formulated to tackle the country’s PE burden.

## Introduction and background

The literature on the morbidity and mortality rates related to myocardial infarction (MI) and stroke is abundant, but there is a significant gap in the epidemiology of pulmonary embolism (PE). PE remains a significant global public health concern, ranking as the third most common cause of cardiovascular (CV) disease mortality worldwide, resulting in approximately 100,000 fatalities annually. In the context of India, it holds the second position in terms of mortality rates, trailing closely behind MI [1,2]. Within Western societies, the occurrence rate stands at one case of deep vein thrombosis (DVT) and 0.5 cases of PE per 1000 individuals annually [3]. In the realm of scientific literature, it is conceivable that mortality rates could substantially exceed the figures indicated by the available data. This is primarily due to a significant portion of PE cases going unreported, being underdiagnosed, or being misdiagnosed as acute MI or ventricular arrhythmias [2,4]. The epidemiology of PE in India is largely ambiguous [5]. In their study, Chaudhary et al. reported a PE prevalence of 14% among individuals with acute exacerbation of chronic obstructive pulmonary disease (AECOPD) [1]. In addition, an autopsy study conducted at a tertiary care center in northern India identified PE as the primary cause of mortality in 16% of hospitalized patients [5,6]. In India, the challenges are compounded by factors such as limited access to advanced diagnostic tools, varying levels of healthcare infrastructure, and a lack of awareness about PE among healthcare providers and the public [3]. Addressing these challenges is vital to reducing the mortality rate associated with PE in India and improving health outcomes on a global scale.

The gamut of presenting symptoms of PE ranges from incidental findings on imaging to the rapid onset of pleuritic chest discomfort, hypoxia, dyspnea, collapse, and death [1]. This condition, commonly known as “the great mimicker” because of its non-specific clinical presentation, continues to be one of the most frequent avertable causes of hospital mortality around the world, accounting for 10% of all hospital deaths [2]. The symptoms of PE often overlap with those of other conditions, which complicates early detection. Given its tendency to mimic several disease entities that impact both the CV and respiratory systems, it is important to recognize the clinical features and risk factors of PE at the earliest. Timely risk stratification aids in treating this illness in the most time-effective and prudent manner while restricting the risk of complications and death [2,4]. Early detection of the disease is also essential because most acute PE fatalities transpire within the initial hours to days, with more than 70% taking place within the very first hour [4].

The risk factors for PE are comprehensively outlined in Table 1, drawing on data from three major registries: the Emergency Medicine Pulmonary Embolism in the Real-World Registry (EMPEROR) [7], the International Cooperative Pulmonary Embolism Registry (ICOPER) [8], and the Registro Informatizado de la Enfermedad TromboEmbólica (RIETE) [9]. These registries provide a detailed overview of various factors that increase the likelihood of developing PE, serving as a critical resource for understanding and managing this condition.

**Table 1:** Risk factors of PE based on the EMPEROR, ICOPER, and RIETE registries BMI: Body mass index; CHF: Congestive heart failure; COPD: Chronic obstructive pulmonary disease; DVT: Deep vein thrombosis; EMPEROR: Emergency Medicine Pulmonary Embolism in the Real-World Registry; HF: Heart failure; ICOPER: International Cooperative Pulmonary Embolism Registry; PE: Pulmonary embolism; RIETE: Registro Informatizado de Enfermedad TromboEmbólica.

EMPEROR (n=1880) [7]		ICOPER (n=2454) [8]		RIETE symptomatic massive PE (n=248) [9]	
Risk factors	Percentage (%)	Risk factors	Percentage (%)	Risk factors	Percentage (%)
Obesity	26.9	DVT	49.3	Age ≥75 years	65
Recent hospitalization	23.8	BMI (>29 kg/m^2^)	29	Cancer	34
Malignancy, active	22.3	Surgery within 2 months	29	Cardiac/respiratory disease	29.2
Current smoker	17.7	Bed rest >5 days	28	DVT	19.2
Recent surgery	14.4	Previous DVT/PE	25	BMI ≥30 kg/m^2^	16
Prior DVT	11.9	Cancer	22.5	Immobilization >4 days due to neurological disease	11
Immobility	11.6	Current cigarette smoking	18	Recent surgery	8
Current DVT	9.5	COPD	12.4		
Family history of DVT or PE	8.4	CHF	10.5		
History of HF	7.5	Central venous catheter	8		
Venous catheter	5				
Use of oral contraceptives	4.4				

Given the significant health burden of PE in India, characterized by its high mortality rates, ambiguous epidemiology, and the challenge it poses due to its nonspecific symptoms, this study aims to fill the existing gap in the literature by providing a comprehensive narrative review of the epidemiology, challenges, and recommendations specific to the Indian context. By synthesizing current evidence and identifying key areas for improvement in diagnosis, treatment, and prevention, this review seeks to offer actionable recommendations to enhance PE management and outcomes in India.

## Review

Methodology

To comprehensively understand the epidemiology and challenges of PE in India, we employed a systematic search strategy. This strategy was designed to capture a broad spectrum of relevant literature.

The databases searched included PubMed, Scopus, Web of Science, Google Scholar, and Cochrane Library for studies published up to May 2023. Keywords and phrases utilized in the search encompassed pulmonary embolism”, “DVT”, “venous thromboembolism”, “oral anticoagulants”, “non-vitamin K oral anticoagulants”, “novel oral anticoagulants”, “vitamin K oral anticoagulants”, “dyspnoea”, “computed tomography pulmonary angiogram” (CTPA), “pulmonology”, “V/Q scan”, “pulmonary angiography”, “India”, “PE challenges”, “risk factors”, and “public health”. These terms were used in various combinations and adjusted as per the specific database syntax requirements. Additionally, we reviewed the reference lists of key articles to identify any additional studies that may not have appeared in the database searches.

Both observational and interventional studies conducted in the Indian context, review articles, case reports, and guidelines were considered for inclusion. The search was restricted to articles published in the English language. The selected articles were then critically appraised for quality and relevance to the review’s objectives. Data extraction focused on the key findings related to the epidemiology of PE in India, diagnostic and management challenges, and proposed recommendations.

This review adheres to the principles of a narrative synthesis, facilitating a comprehensive understanding of the subject matter through thematic analysis of the gathered literature.

Clinical characteristics of PE

Presenting Symptoms and Clinical Features of PE

The clinical features of PE are often ambiguous and detected incidentally during the diagnostic evaluation of another ailment [10]. Although PE may present with a wide spectrum of nonspecific manifestations, the most typical symptoms include cough, dyspnea, pleuritic chest discomfort, delirium, abdominal pain, and fever [11,12]. The clinical features of PE are enumerated in Table 2.

**Table 2:** Clinical presentation of PE documented from various registries/studies EMPEROR: Emergency Medicine Pulmonary Embolism in the Real-World Registry; ICOPER: International Cooperative Pulmonary Embolism Registry; PIOPED: Patients in the Prospective Investigation of Pulmonary Embolism Diagnosis

Symptoms	EMPEROR [7]	ICOPER [8]	PIOPED II [13]	Davidsingh et al. [14]
Dyspnea	50%	82%	73%	91.4%
Tachypnea		60%	57%	23%
Tachycardia		40%	26%	
Chest pain	54%	49%	76%	17%
Upper abdominal pain	11%			
Syncope	6%	14%		
Cough	23%	20%	44%	4%
Hemoptysis	8%	7%	6%	
Respiratory distress	16%			

Differential Diagnoses of PE

The presence of negative T waves in the electrocardiogram (ECG), a classic feature of acute coronary syndrome (ACS), is frequently observed in PE, especially in individuals who are susceptible to adverse consequences [15]. Several symptoms of PE, such as dyspnea and chest pain, can be challenging to discern from those of ACS. This often leads to misdiagnoses of PE as ACS, which leads to avoidable deaths in individuals with PE [15]. A history of asthma may occlude the diagnosis of PE, as PE can also present with asthma-like symptoms “asthmatic crisis”. Therefore, the investigations for PE should comprise appropriate ECG, chest radiography, and blood tests to form a conclusive diagnosis [12]. PE must be differentiated from pneumonia when presented with fever and displayed evidence of pulmonary infiltrates on the radiograph [10,12]. PE should be included in the list of potential differential diagnoses for individuals who present with syncope (particularly with those accompanying dyspnea, respiratory distress, or hypoxemia). Recent studies have shown syncope frequency in those with high-risk PE to be ranging between 29.9% and 35% [12,16].

Diagnostic methodologies and misdiagnosis of PE

Diagnostic Approaches to PE

PE falls within the spectrum of venous thromboembolic (VTE) diseases, typically triggered by blood clots originating in the deep veins of the femoral, popliteal, and iliac regions. The reliance on generic clinical investigations such as arterial blood gas analysis, chest radiographs, and ECG, which possess low sensitivity and specificity, significantly complicates the diagnosis of PE [2,3].

The CTPA is presently the standard of care and serves as a standalone imaging test in the diagnosis of PE. It has a rapid turnaround time, and the Prospective Investigation of Pulmonary Embolism Diagnosis (PIOPED II) study observed sensitivity and specificity of 83% and 96%, respectively, for CTPA in PE diagnosis [10,13].

Pulmonary angiography represents the diagnostic gold standard for PE, but this technique is expensive, invasive, difficult to obtain, and labor-intensive [17]. Catheter pulmonary angiography is currently employed exclusively for interventional care of PE and is no longer utilized as a diagnostic tool. Chest radiography has limited efficacy as it sporadically displays outcomes of PE or infarction but is useful in dismissing various other possible reasons for chest discomfort. The ventilation-perfusion (V/Q) scan demonstrates V/Q disparities in individuals exhibiting symptoms suggestive of PE. These variations are categorized by numerous classification systems, ranging from normal to high. Despite its diagnostic value, the V/Q scan is burdened by challenges related to its availability and time-consuming nature, rendering it generally unsuitable for emergent or urgent clinical scenarios. Nevertheless, it serves as a valuable alternative when CTPA is either unavailable or contraindicated. Although only available in specialized centers and requires a higher degree of competence, magnetic resonance imaging (MRI) offers accurate diagnosis. Echocardiography is used for risk stratification among PE-suspected patients [5,10,18-20].

Chest radiography, ECG, echocardiogram, V/Q scan, CTPA, and laboratory tests of cardiac strain (D- dimer and troponin) are commonly used diagnostic tools in Indian settings [1,6,14,21]. However, PE should not be suspected and investigated in every patient presented with dyspnea and chest pain. To avoid unnecessary investigations, the PE rule-out criteria (PERC) was developed. The PERC comprises eight clinical parameters that are linked to the absence of PE, namely: age under 50 years, pulse rate below 100 beats per minute, oxygen saturation level above 94%, absence of unilateral lower limb swelling, absence of hemoptysis; no recent history of trauma or surgical procedures, no previous occurrence of VTE, and non-usage of oral contraceptives [10].

**Figure 1:**
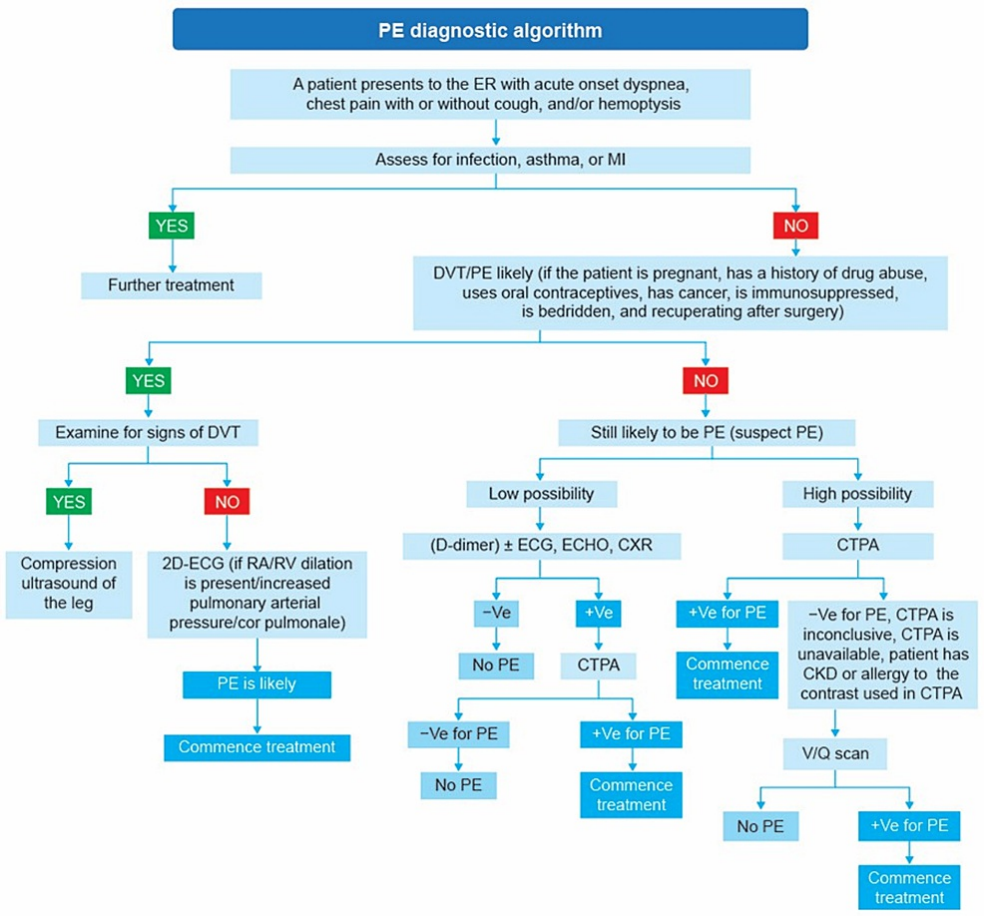
Diagnostic algorithm for PE. CTPA: computed tomography pulmonary angiography; CXR: chest radiograph; DVT: deep vein thrombosis; ECG: electrocardiogram; ER: emergency room; MI: myocardial infarction; PE: pulmonary embolism; RA: right atrium; V/Q scan: ventilation (V) perfusion (Q) scan; -Ve: negative; +Ve: positive; 2D: two dimensional The image is created by the authors of this article.

To simplify the diagnosis/management of PE, the PE response teams (PERTs) were conceived to improve efficiency, decision-making, and patient access to advanced therapies. A PERT is composed of clinicians from a range of specialties, including emergency medicine, internal medicine, obstetrics and gynecology, surgical services, pulmonology, critical care, interventional radiology, cardiology, vascular and cardiothoracic surgery, among others [10,22,23]. A probable diagnostic algorithm for PE is illustrated in Figure 1.

Misdiagnosis of PE

PE is the second most misdiagnosed condition in a hospital setting [18]. The most common misdiagnoses of PE are pneumonia, bronchitis, COPD, HF, and ACS. Out of all the misdiagnosed conditions, the proportion of a false positive for pneumonia, bronchitis, or COPD was 37.4% whereas the percentages of false positives for HF and ACS were 18.2% and 12.4%, respectively [18]. The nonspecific nature of PE symptoms complicates accurate diagnosis. Research suggests that the infrequent reporting of PE misdiagnoses may be attributed to this challenge [9,18]. PE misdiagnosis can have serious consequences, as it can prolong hospital stays, subject patients to needless therapies, and cause them to deteriorate as a result of waiting too long for the right care [2,12,24].

PE as a comorbidity

PE in Patients with Acute Exacerbation of Chronic Obstructive Pulmonary Disease (AECOPD)

AECOPD significantly increases morbidity, healthcare utilization, and mortality. Various studies have confirmed AECOPD as an independent predisposing factor for PE (the overall PE prevalence in AECOPD was 16% in a systematic review and meta-analysis). Additionally, patients with AECOPD and PE have greater 3-month and 1-year mortality rates compared to patients with PE alone [1].

PE and Chronic Kidney Disease (CKD)

Patients with CKD are frequently hospitalized and in a procoagulant state (increased plasma concentrations of procoagulant proteins like prothrombin and fibrinogen are recognized risk factors of thrombosis). As a result, those with CKD are more susceptible to VTE and PE [21].

PE in Postoperative Patients

PE is a significant contributor to mortality among postoperative patients. The risk of fatality is notably elevated following major surgical procedures involving the abdomen, pelvis, or lower extremities. However, due to the generic clinical symptoms that are readily missed, PE in postoperative patients is still substantially underdiagnosed. Studies have established that prediction models aid in making prompt diagnoses and anticoagulants can be used as a prophylactic strategy after surgery [25].

Management of PE

PE can cause abrupt right ventricular (RV) failure and a complex chain of events that result in rapid hemodynamic and respiratory collapse. Supportive treatment of PE is critical in such cases, which can be accomplished by oxygen therapy, ventilator support, volume expansion therapy, mechanical pulmonary breathing (invasive and noninvasive), pharmacological CV maintenance, RV function sustenance, and administration of bronchodilators and antibiotics [26].

In essence, the PE treatment strategy should always consist of three major components, which are described in Table 3.

**Table 3:** Treatment approach for PE CDT: catheter-directed thrombolytic; ECMO: extracorporeal membrane oxygenation; LMWH: low-molecular-weight heparin; LV: left ventricle; NOAC: non-vitamin K oral anticoagulants; OAC: oral anticoagulant; PA: pulmonary artery; PE: pulmonary embolism; RV: right ventricle

Treatment approach	
Cardiopulmonary support	It is instigated through supplemental oxygen therapy, vasopressors, inotropes, and preload. In the event in which aforementioned treatments are unsuccessful in treating the RV, more aggressive concurrent procedures, including surgery or ECMO, should be started [4,27].
Anticoagulation	Treatment with heparin (unfractionated or LMWH), OACs, and more recently, the NOACs, should be initiated promptly when PE is suspected, unless there is a clear contraindication for its use [4,28].
Reperfusion of the PA	Reperfusion treatment is introduced to avoid cardiogenic shock and circulatory arrest. It is achieved through - systemic thrombolysis: This is the most frequently employed method and has been demonstrated to reduce mortality rates by up to 2.4% in patients with intermediate and massive PE [4,29]. CDT: It is used to treat massive and submassive PE with hemodynamic instability. It is an alternative procedure to systemic thrombolysis among patients with a high risk of bleeding [4]. In the SEATTLE II study, CDT successfully attained a 25% reduction in the diameter of the RV/LV, a 30% decrease in PA blockage, and a 30% decrease in PA systolic pressure [30]. Surgical pulmonary thrombo-embolectomy: It is a therapeutic option among patients with RV dysfunction, as it can immediately alleviate the burden on the RV and pause the development of cardiogenic shock. There is a greater mortality risk associated with surgery, which could be because those undergoing the procedure are, by definition, at an elevated risk compared to those who undergo nonsurgical interventions [4]. An alternative percutaneous technique involves clot aspiration through the use of a specialized suction embolectomy device. This method offers the advantage of swiftly removing the thrombus without raising the bleeding risk linked to thrombolysis [31].

Non-vitamin K Antagonist Oral Anticoagulants

Anticoagulation therapy is the fundamental aspect of managing PE, with guideline recommendations on therapy extending up to 3 months or longer [4,28,29]. Every patient who has a high clinical likelihood of developing PE should be prescribed anticoagulants as soon as feasible (preferably during the diagnostic process or prior) [26]. If the risk-benefit ratio between the probability of PE and the bleeding risk appears favorable, anticoagulation should be instituted even before the diagnosis of PE is confirmed. Once the diagnosis is established, risk stratification and triage are critical to understand which patients might benefit from an intrahospital transfer [26]. Conventional anticoagulation therapy is based on intravenous unfractionated heparin or low-molecular-weight heparin (LMWH) typically followed by vitamin K antagonists (VKAs), including oral anticoagulants such as warfarin [26,29]. This regimen is challenging despite being effective because it requires daily subcutaneous injections of heparin/LMWH and frequent dose monitoring and adjustment, especially in the case of VKA, i.e., warfarin therapy [28,29]. Over the past few years, new and emerging oral anticoagulants, referred to as “non-vitamin K antagonist oral anticoagulants” (NOACs), also known as direct oral anticoagulants (DOACs), comprising apixaban, edoxaban, rivaroxaban (factor Xa inhibitors), and dabigatran (direct thrombin inhibitor) were developed to overcome the limitations of warfarin and its analogs [32]. The various characteristic features of NOACs are demonstrated in Table 4 [33,34].

**Table 4:** Properties of NOACs BID: twice daily; mg: milligram; NOACs: non-vitamin K antagonist oral anticoagulants; OD: once daily

Characteristic	Dabigatran [33,34]	Rivaroxaban [33,34]	Apixaban [33,34]	Edoxaban [33,34]
Target	Factor IIa	Factor Xa	Factor Xa	Factor Xa
Renal clearance (%)	80	66	27	50
Bioavailability (%)	6.5	>80	50	62
Metabolism (% of liver metabolism)	20	66	70	50
Half-life (h)	14-17	5-9 (young) 11-13 (elderly)	10-14	10-14
Protein binding (%)	34-35	92-95	87	55
Peak effect (h)	2	2-4	3-4	1-2
Antidote	Idarucizumab	Andexanet - α	Andexanet - α	Andexanet - α
Full dose	150 mg BID	20 mg OD	5 mg BID	-

**Figure 2:**
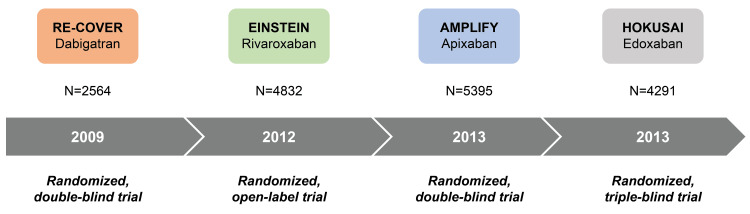
Timeline of landmark NOAC trials. NOAC: Non-vitamin Kk antagonist oral anticoagulants The image is created by the authors of this article.

The major trials conducted to compare NOACs with conventional anticoagulants in treating PE/VTE were the RE-COVER [35], EINSTEIN [36], AMPLIFY [29], and HOKUSAI [37] trials (Figure 2).

NOACs represent an archetypical shift in the treatment of cardiopulmonary disorders. With their predictable pharmacodynamic effects and pharmacokinetic profile, NOACs were developed to offer efficient anticoagulation while eliminating the need for monitoring. They possess an expeditious onset of action, predictable half-life, less intracranial bleeding, and rare drug-drug and food-drug interactions [32,38,39]. NOACs have the added benefit of a safe transition from VKAs and a similar rapid onset of action to LMWH with oral administration. This is unlike traditional anticoagulant therapy with VKAs including the numerous food and drug interactions, the requirement for international normalized ratio (INR) monitoring, and the need to alter doses, even though it is effective and safe [40]. Therefore, NOACs have emerged as an attractive alternative to VKAs in the management of PE. The universal concerns/queries regarding the usage of NOACs for PE are exemplified in Table 5 [38,41-47].

**Table 5:** Treatment and follow-up of PE using NOACs BID: twice a day; DVT: deep vein thrombosis; HoT-PE: home treatment of patients with low-risk pulmonary embolism; NOAC: nonvitamin K antagonist oral anticoagulant; OD: once a day; PE: pulmonary embolism; PESI: pulmonary embolism severity index; VKA: vitamin K antagonist; VTE: venous thromboembolism; WHITE: WHIch Decision After a First Venous ThromboEmbolism

Case settings	Approach
Early hospital discharge and home-based treatment of individuals at low risk of mortality due to PE (ambulatory treatment of PE)	Research indicates that apixaban and rivaroxaban provide a practical single-drug treatment strategy, which does not hinder the patient’s lifestyle and domestic responsibilities. Moreover, the NOACs are distinguished by uncomplicated oral administration, absence of regular coagulation monitoring, rapid absorption rate, 12-hour half-life, and 25% renal excretion [38]. Numerous phase III clinical trials have provided strong evidence regarding the comparable effectiveness and enhanced safety profile of NOACs, such as apixaban and rivaroxaban. These medications differ in terms of dosing schedules, with apixaban requiring a 7-day regimen of 10 mg BID and rivaroxaban necessitating a 21-day regimen of 15 mg BID for high-intensity anticoagulation [41]. Due to the above reasons, NOACs can be prescribed during early discharge and treatment in an outpatient setting for low-risk patients, thus alleviating the pressure on emergency care resources. The HoT-PE trial affirms the same, provided there is a validated criterion (such as PESI and its simplified version sPESI) for selecting only low-risk PE patients. This was recommended on the grounds of medical, ethical, and legal concerns. Although NOACs are relatively safe, it is critical to inform newly discharged patients about NOAC medication, the possible hazards of bleeding from NOAC use, and the options for management in case of bleeding episodes. Moreover, the decision for domiciliary treatment of PE should only be made following a comprehensive evaluation of the patient’s risk factors and their ability to promptly access specialized healthcare if the need arises [42-44].
Extension treatment in patients with provoked and unprovoked DVT	According to the WHITE study, for over a decade, international guidelines recommend anticoagulants for a period of 3–6 months in DVT if provoked by surgery or another specific removable risk factor [45]. The guidelines recommend an extended indefinite period of anticoagulation when the risk factors are permanent (such as cancer, inflammatory diseases, or repeated VTE events) and in case of unprovoked DVT with no apparent risk factor. The extended period of anticoagulation is also based on the extended therapy risk-benefit ratio after 3 months [45,46].
Long-term anticoagulant treatment (>3 months) for all patients with PE	Anticoagulants are given initially to prevent the extension of the thrombus and later in long-term therapy to prevent the relapse of PE and death. The recent evidence-based guidelines also endorse long-term oral anticoagulation of indefinite duration for select individuals who are considered to be at high risk for recurrence of VTE. This would include patients who have developed a VTE without any apparent risk factors or those with minimal, temporary, or reversible risk factors [41]. VKA or NOACs can be used for long-term anticoagulation treatment in PE patients who need a longer maintenance phase [41]. Once the long-term anticoagulation (with NOAC) is planned, the guideline-recommended dosage is apixaban (2.5 mg BID) or rivaroxaban (10 mg OD) after 6 months of anticoagulant therapy [47].

Management of PE in Patients with Cancer

PE is a common cause of death and morbidity in cancer patients. Management of cancer patients with VTE is complicated compared to non-cancer patients due to the higher risk of recurrent thromboembolism, bleeding, and the cancer therapy the patient is on. The management also depends on whether the cancer is currently in an active state or if there exists a past medical record of cancer. Although some guidelines have recommended LMWH for the initial treatment of cancer-associated VTE/PE (after the first 3-6 months, LMWH is substituted with oral anticoagulants), and of late, NOACs have emerged as an effective and more acceptable alternative in anticoagulation treatment in cancer patients [47,48]. The primary outcomes from trials comparing NOACs with other anticoagulants among cancer patients with PE are illustrated in Table 6.

**Table 6:** Characteristics and outcomes from clinical trials assessing the treatment of PE in individuals with cancer CRNB: clinically relevant nonmajor bleeding; DOAC: direct oral anti-coagulants; GI: gastrointestinal; LMWH: low-molecular-weight heparin; s.c: subcutaneous; VTE: venous thromboembolism

Trial name	Study design	Intervention	Outcome measures	Results	Inference
Caravaggio study [48,49]	Randomized, open-label, non-inferiority trial	Oral apixaban or s.c dalteparin	Recurrent VTE and major bleeding	Oral apixaban exhibited non-inferiority when compared to subcutaneous dalteparin, showcasing favorable efficacy and safety profiles, along with the absence of an elevated risk of serious bleeding.	There was no observed elevation in GI bleeding among patients treated with apixaban, including those with gastrointestinal cancer.
SELECT-D trial [50]	Multicenter, randomized, open-label, pilot trial	Oral rivaroxaban or s.c dalteparin	Recurrent VTE	Rivaroxaban was linked to lower recurrent VTE but a higher rate of CRNMB, when compared with dalteparin.	Bias could have been introduced as it was a pilot study and amendments were made during the trial. The trial lacked sufficient statistical power.
Hokusai-VTE cancer study [51]	Randomized, open-label, non-inferiority trial	LMWH, followed by oral edoxaban or s.c dalteparin	Recurrent VTE and major bleeding	Oral edoxaban demonstrated comparable efficacy to subcutaneous dalteparin in the event of recurrent VTE in patients with various types of cancer.	Edoxaban demonstrated a reduced incidence of recurrent VTE compared to dalteparin, while it displayed a higher occurrence of major bleeding.
CANVAS trial [52]	Hybrid non-inferiority trial, with randomized and preference cohorts.	Any DOAC or LMWH	Recurrent VTE	DOACs had a non-inferior risk of recurrent VTE and no difference in bleeding, mortality, or serious adverse events, compared to LMWHs.	The unblinded trial design may have introduced bias to the study results.

The results of the trials indicate that NOACs show potential as a viable alternative to LMWH in the management of cancer-associated VTE in patients with various types of cancer. The National Comprehensive Cancer Network (NCCN) recommends the evaluation of NOACs as an option in treating PE associated with cancer albeit, it is important to exercise caution when considering the treatment with patients who have gastrointestinal cancer [53].

Need for a unified PE guideline in India

A study by Muralidharan et al. concluded that PE presented in India, 10 years earlier than similar events in Western countries and has been linked to unfavorable clinical results (2.3% higher than in the West) [6]. The data on the treatment and associated complications of PE in India are scarce, and the vast majority of the evidence is based on case reports and a limited number of small-scale investigations [14]. Research further suggests that Indians exhibit a stronger propensity for the development of PE at a much earlier age in comparison to the global demographic trends. The mortality rate of PE is also higher in India [6]. Through their studies, Muralidharan et al. [6] and Davidsingh et al. [14] established the average age of Indians who have PE as 50 years and 52 years respectively as opposed to 65 years and above in the West. The Arrive registry reiterated the aforementioned data [6].

There is a wide gap in the statistics of PE in India and a unified diagnostic and management guideline for the country is not yet formulated. One major reason for this could be the scarcity of records, and the other reason could be the sociodemographic and socioeconomic makeup of the country. The gold standard diagnostic test of PE-the CTPA-is not available in many healthcare centers in India. To overcome this predicament, the awareness about PE among healthcare professionals in India should be increased for diagnosing the disease even in the absence of contemporary diagnostic tests (using scoring criteria such as the Wells score, Geneva score, or the PERC) [54].

## Conclusions

Although PE is a fatal disease and can be managed effectively if identified at an earlier phase, mortality and morbidity rates of the disease are still high in India. This could be due to the dearth of data on the prevalence and incidence of the disease in India. The majority of Indian data are based on sporadic stand-alone studies and postmortem findings. If an accurate depiction of PE in India is acquired, a unified pan-country guideline and approach can be formulated for tackling the disease burden.
